# Genetic model misspecification in genetic association studies

**DOI:** 10.1186/s13104-017-2911-3

**Published:** 2017-11-07

**Authors:** Amadou Gaye, Sharon K. Davis

**Affiliations:** Metabolic, Cardiovascular and Inflammatory Disease Genomics Branch, Social Epidemiology Research Unit, National Institutes of Health, National Human Genome Research Institute, Bethesda, USA

**Keywords:** Genetic association analysis, Incorrect genetic model, Statistical power

## Abstract

**Objective:**

The underlying model of the genetic determinant of a trait is generally not known with certainty a priori. Hence, in genetic association studies, a dominant model might be erroneously modelled as additive, an error investigated previously. We explored this question, for candidate gene studies, by evaluating the sample size required to compensate for the misspecification and improve inference at the analysis stage. Power calculations were carried out with (1) the true dominant model and (2) the incorrect additive model. Empirical power, sample size and effect size were compared between scenarios (1) and (2). In each of the scenarios the estimates were evaluated for a rare (minor allele frequency < 0.01), low frequency (0.01 ≤ minor allele frequency < 0.05) and common (minor allele frequency ≥ 0.05) single nucleotide polymorphism.

**Results:**

The results confirm the detrimental effect of the misspecification error on power and effect size for any minor allele frequency. The implications of the error are not negligible; therefore, candidate gene studies should consider the more conservative sample size to compensate for the effect of error. When it is not possible to extend the sample size, methods that help mitigate the impact of the error should be systematically used.

**Electronic supplementary material:**

The online version of this article (10.1186/s13104-017-2911-3) contains supplementary material, which is available to authorized users.

## Introduction

In genetic association studies the underlying genetic model of inheritance of the genetic determinant of a trait is not always known with certainty [1–3]. It is however known that in general when mathematically correct, and for similar minor allele frequency (MAF) and effect size, additive genetic models provide more power than dominant ones [4]; we are talking here about complete dominance where one copy of the dominant allele is sufficient to reach the full effect. For the remainder of this document we refer to such dominant model as ‘binary’ model and name the variant under this model a binary variant to convey the idea that, under complete dominance, there are only two genotype groups.

In genetic association studies, it is common practice to assume an additive model when the model of the genetic determinant of interest is unknown [1]. It is, hence, plausible that in some studies a binary genetic variant is incorrectly modelled as additive. This represents a misspecification of the true underlying genetic model, an error which could have an adverse effect on the statistical power of an association [5, 6] and on the effect size. The genotype of an individual at a locus is the combination of the alleles on each of the two homologous chromosomes. If the two alleles are combined incorrectly the individual might be assigned an incorrect genotype. This can lead to genotypes misclassification but the mechanism we describe is not in itself genotype misclassification as described by Hossain et al. [7]; what we are referring to is the fact that alleles are not combined using the true genetic model.

This issue has been previously studied and some solutions proposed [1, 6, 8–17]. Therefore, rather than proposing a new method it is probably more important to explore this question from a less technical angle to incite a change in the practice (i.e. researchers to consider other possible models, if feasible). We explored the effects of genetic model misspecification on statistical power, sample size and effect size in candidate gene association studies with the aim of evaluating the impact of the error in a hypothetical study where the outcome is either a binary or a continuous trait. We are interested in estimating the sample size required to compensate for the misspecification and improve inference at the analysis stage.

**Fig. 1:**
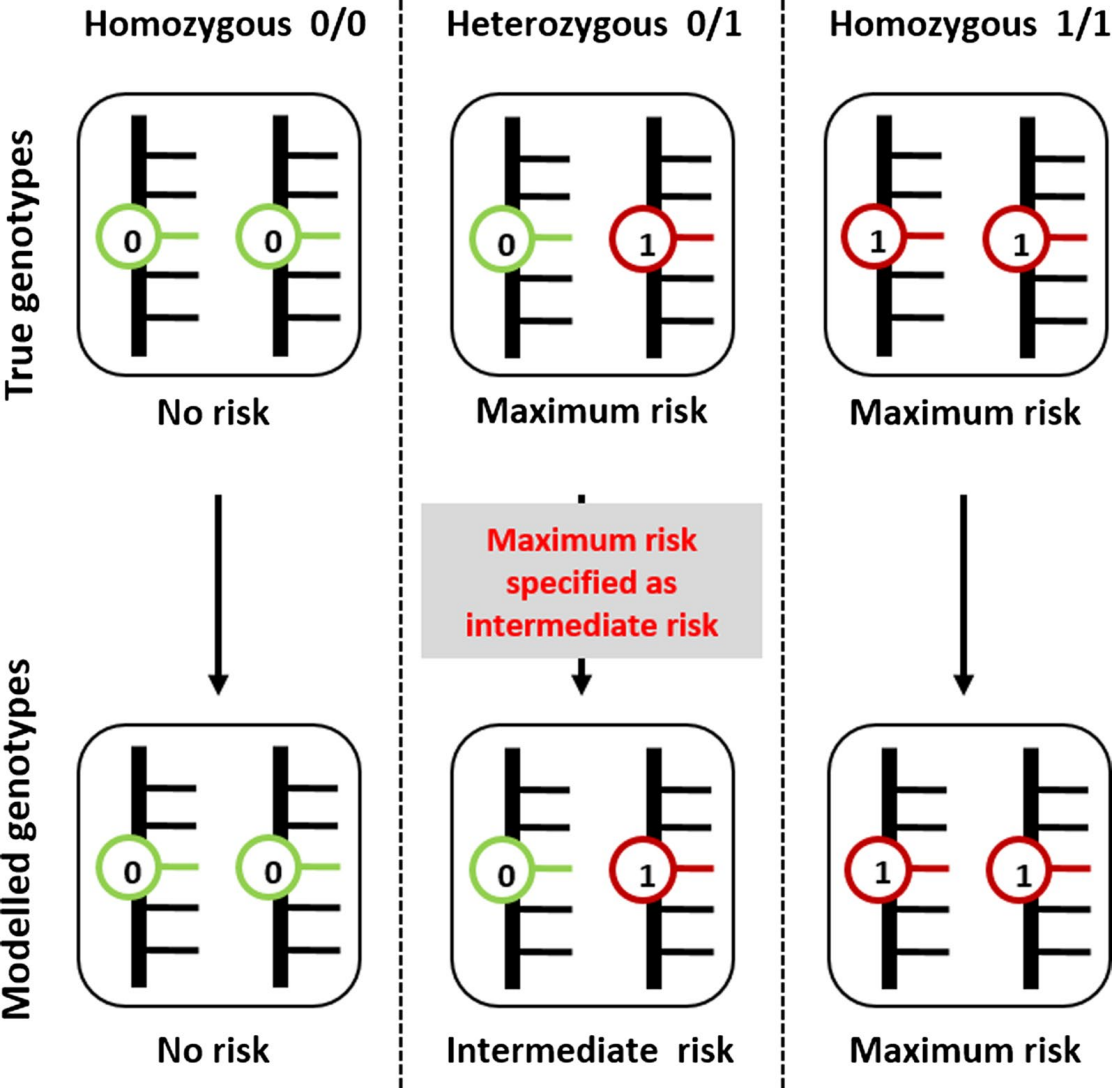
This illustration assumes a bi-allelic SNP. If a binary model is analysed as additive the risk is underestimated for heterozygous individuals whose risk is half the actual true risk

We considered a bi-allelic single nucleotide polymorphism (SNP) and focused on an underlying model where the allele that confers the risk is in complete dominance (Fig. 1).

## Main text

### Methods

Power and sample size calculations were carried out using the ESPRESSO calculator developed from our earlier work [18]. This tool has been already used by Gaye et al. [19] to analyse the impact of pre-analytical variation on power in the UK Biobank. We used ESPRESSO because unlike closed form solutions proposed by conventional calculators, it allows for the flexibility required to include uncertainties around outcome and covariates in power calculations.

#### Brief apercu of an ESPRESSO simulation process

An ESPRESSO process involves repeatedly simulating a dataset with key characteristics and evaluating in what proportion of the simulations the effect of interest can be detected by an appropriate method of statistical inference at a given level of statistical significance. The purpose of this section is to explain succinctly how the exposure and outcome data are generated and the association evaluated.

The exposure/genotype data are generated by first simulating the two alleles, a wild allele denoted 0 and the allele that confers the risk (referred to by ‘risk allele’ from now on) denoted 1.

$$Allele \sim B(n, MAF)$$

n = number of observations, MAF = minor allele frequency

The genotypes are obtained by combining the alleles based on the underlying genetic model. If the underlying model is binary there are only two genotypes: 0 and 1 (since heterozygous, 0/1, and homozygous, 1/1, individuals are at the same risk level). And if the underlying genetic model is additive the genotypes of homozygous, heterozygous and homozygous 1/1 individuals are coded respectively as 0 (0 + 0), 1 (0 + 1) and 2 (1 + 1).

The binary outcome data is generated by first obtaining a linear predictor (LP) and then using the expit transformation of the LP to compute the probability of disease (mu). The binary outcome (OUT_BINARY_) is hence a binomially distributed random variable with a probability mu.

LP = β_0_ + β_1_G_1_ + s.effect where $\varvec{ }\beta_{0} = log\left( {\frac{disease prevalence}{1 - disease prevalence}} \right)$ and G_1_ is the exposure data and β_1_ is the odds-ratio associated with the exposure

$$mu = \frac{{e^{LP} }}{{1 + e^{LP} }}\;\;\;\;\;\;\;\;\;\;\;\;\;\;OUT_{BINARY} \sim B(n, mu).$$

For the binary outcome, there is an additional term, the subject effect (*s.effect*), which reflects the heterogeneity in disease risk arising from determinants not measured or not included in the model. This effect is computed from the baseline OR parameter. When we set the baseline OR to 10 as in our simulation, we are essentially saying that given all the un-measured parameters an individual at high risk (one in the top 95% of population risk) has, all else being equal, odds of developing the disease that are 10 times that of a person considered to be at low risk (one in the bottom 5% of population risk). The variance in baseline risk $\varvec{ }\sigma_{baseline.OR}^{2}$ for an individual on the 95th vs. 5th population percentile, is assumed to follow a normal distribution on the logistic scale. It hence must be converted into the corresponding variance, for a normally distributed effect $\varvec{ } \sigma_{subject.effect}^{2}$; the conversion is carried out using the below equation where ***z***_**0.95**_ is the z-score associated with 95 percentile.

$$\varvec{ }\sigma_{subject.effect}^{2} = \left[ {\frac{{\sigma_{baseline.OR}^{2} }}{{2 \times z_{0.95} }}} \right]^{2} \;\;\;\;\;\;\;s.effect \sim N(0, \sigma_{subject.effect}^{2} )$$

The subject effect for an individual is drawn from a normal distribution with a mean of zero and a variance $\sigma_{subject.effect}^{2} .$ The variance in baseline risk is a normally distributed error term that is added to the linear predictor. Both this distribution and the specified magnitude of the parameter are inevitably arbitrary but a sensitivity analysis can be undertaken and, in most settings, the required sample size is remarkably robust to variation in the specified baseline OR.

The continuous outcome (OUT_CONTINUOUS_) is a normally distributed random variable with a mean LP and a given standard deviation.

$$OUT_{CONTINUOUS} \sim N\left( {LP, \sigma } \right)$$

The association is assessed by fitting the appropriate generalized linear model (GLM) and using a large sample z-statistic to test the null hypothesis of no genetic association.

Estimating sample-size and power in R by exploring simulated study outcomes can be used to calculate (A) the sample required to achieve a desired power and (B) estimate the power that can be achieved with a given sample size. In this work both features of the tool were used. For each of (A) and (B), the calculations were carried out twice, as outlined in section 1 of the Additional file 1.

### Results

#### Impact of the misspecification error across MAFs

The below two sections report graphically the results of the sample size and power calculation respectively for a binary and a quantitative outcome across rare (MAF ≤ 0.01), low frequency (0.01 ≤ MAF < 0.05) and common SNPs (MAF ≥ 0.05). The results are presented in tabular format in section 2 of the Additional file 1.

**Fig. 2:**
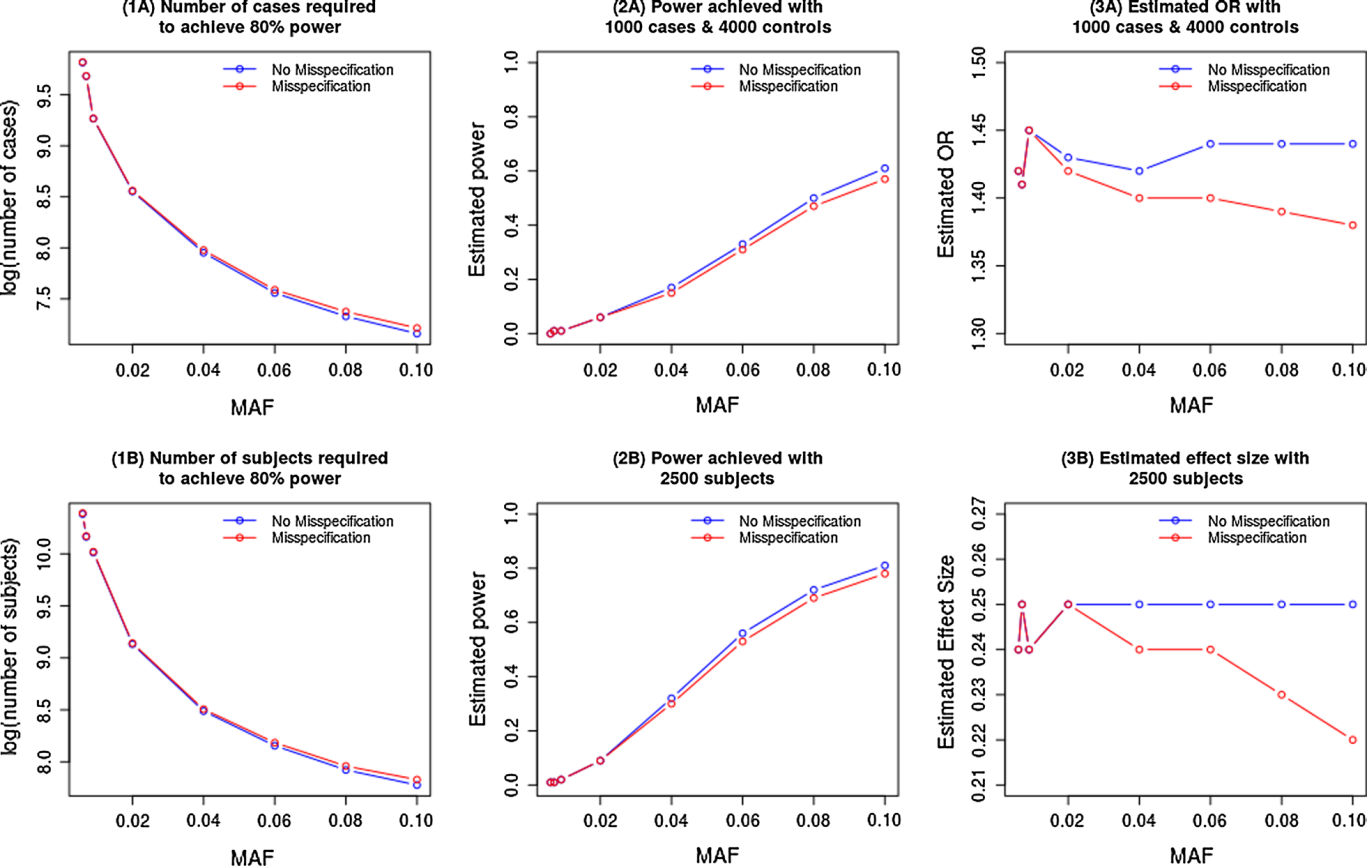
**A** Plots for a binary outcome and **B** plots for a continuous outcome. The sample size required to achieve 80% is lower when the true model is specified (plot 1). The power achieved is higher when the true model is specified (plot 2). And, the shrinkage of the odds-ratio is relatively smaller when the true model is specified (plot 3)

The plots in Fig. 2 (A plots), show that the increase in sample size required to achieve this power with the misspecified model increases with increasing MAF which indicates a greater effect of the misspecification as the risk allele, and incidentally the disease, becomes more prevalent. Under misspecification, power is lower and the odds-ratios shrunk more markedly when the incorrect model is fitted.

The impact on power, shown in Fig. 2 (B plots), is similar to the observations for a binary outcome but the loss of power due to the misspecification is less pronounced. The increase in sample size required to achieve this power with the misspecified model increases with increasing MAF. Under the true model the effect size did not shrink but there is a relatively large shrinkage of the effect size when the true model is not used.

#### Impact of the misspecification on effect size

**Fig. 3:**
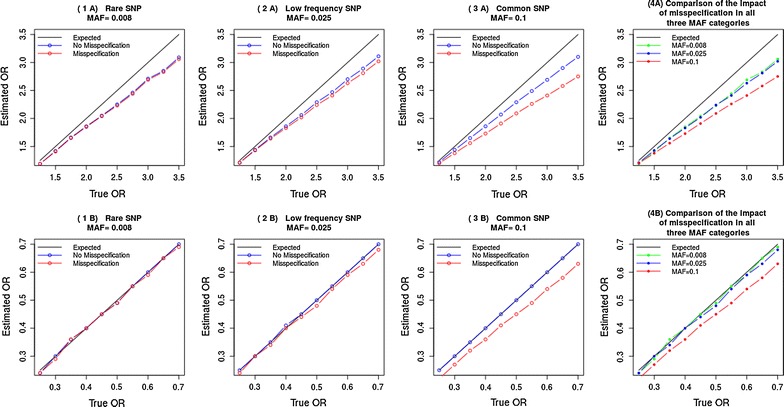
**A** Plots for a binary outcome and **B** plots for a continuous outcome. Estimated odds-ratio for a rare (plot 1), low frequency (plot 2) and common SNP (plot 3). The comparison in plot 4 shows that the odds-ratio is less affected by the misspecification of the underlying genetic model when the SNP is rare

In the results reported above, the misspecification error seems to have a relatively lower impact on the effect size, for rare SNPs. Therefore, we decided to explore the impact of the error across a range of effect sizes. We investigated the effect of the error for a rare (MAF = 0.008), low frequency (MAF = 0.025) and common SNP (MAF = 0.1) across eight effect sizes. The plots in Fig. 3 (A plots) show that, for a binary outcome, the OR is less affected by the misspecification error when the SNP is rare. In Fig. 3 (A plots), the values, for the ‘no misspecification’ scenario, are not overlapping with the ‘expected’ values as one would expect in the absence of error, because the heterogeneity in baseline disease risk that we already mentioned causes some deviation from the true effect size.

Similar observations about the impact of the error, reported above, can be made for a continuous outcome (Fig. 3, B plots).

### Discussion

The analyses show that the misspecification of the true underlying genetic model of a SNP affects the statistical power and the effect size in genetic association studies. The magnitude of the effect of the error, as observed in our results, was however not anticipated.

Power is lost if the genetic model of the SNP is not correctly specified. The loss is relatively small for rare SNPs and larger for low frequency and common SNPs. The adverse effect of the genetic model misspecification on power was more pronounced for an association study with a binary outcome than for a study with a continuous outcome. This is not surprising because, all else being equal, associations with binary outcomes are known to be less powered than those with continuous outcomes [4].

Under a true binary genetic model an individual with two copies of the risk allele (homozygous 1/1) is at the same risk as an individual with only one copy because the additional allele does not increase further the risk while under an additive model the risk for heterozygous (0/1) is half that of homozygous 1/1. Thus, if a binary model is incorrectly specified as additive, the risk for heterozygous individuals is underestimated and this underestimation represents an error that decreases the power of a study. The proportion of heterozygous individuals increases with increasing MAF and hence the proportion of individuals whose risk is underestimated becomes larger; it follows that the magnitude of the error resulting from the genetic model misspecification becomes larger. This explains why the loss of power increases with increasing MAF. Furthermore, we previously stated that under a binary model the calculator assigns the genotype 1 to both homozygous 1/1 and heterozygous individuals so that there are only two genotype classes (0 and 1) but when the genetic model is misspecified homozygous 1/1 individuals are assigned to the genotype class 2 because the two alleles add up; hence some genotypes that should have been 1 are misclassified and such misclassification is also an error that affects the statistical power.

Ensuring the observed effect size is close to the actual true effect size (i.e. no shrinkage towards the null) is extremely important, especially when the genetic studies account for potentially important confounding covariates or gene-environment interactions. If the effect size is underestimated there is a risk of overlooking an association because the observed effect size is not epidemiologically and/or clinically relevant. This alone represents, in our view, a good motivation to investigate the true underlying genetic model before undertaking an association study with a ‘promising’ candidate variant rather than using the current ‘rule of thumb’ of assuming additivity whenever there are no clues about the genetic model of a SNP.

It is clear, from this work that assuming an additive model when an underlying genetic model is not known can lead to an error that affects the power of a study and distort some estimates such as the effect size. For candidate gene studies at the design stage it is worth considering the most conservative sample size, i.e. the sample size that provides sufficient power even when the genetic model is not correctly specified. However, often studies do not have the latitude to extend their sample size; in such cases if no biological clues are available to ascertain the underlying model possible models should be tested to identify the one that better fits the data [6, 20] and the results adjusted for testing multiple models. Furthermore, available methods that implement association tests known to be robust against model misspecification [1, 14] should be used to reduce the impact of the misspecification error.

## Limitations

The analysis did not include investigations of the case assuming a dominant model when the actual model is additive. We also did not consider a recessive model which might not be well studied with the strategy used in this analysis.

## Additional file

**Additional file 1.** Further details about the simulations, parameter settings and results in table format.

## Abbreviations

ESPRESSO: estimating sample-size and power in R by exploring simulated study outcomes
SNP: single nucleotide polymorphism
MAF: minor allele frequency
