# Supplementary material for: Genetic model misspecification in genetic association studies
Source: BMC Res Notes. 2017 Nov 7;10:569. doi: 10.1186/s13104-017-2911-3 (PMC5678796; doi:10.1186/s13104-017-2911-3)
Supplement: Supplementary file 1 — Additional file 1. Further details about the simulations, parameter settings and results in table format. [file 13104_2017_2911_MOESM1_ESM.pdf]

ADDDTIONAL FILE 1

1. Details of the analyses and parameter settings for the simulations

ESRPESSO can be used to simultaneously calculate (A) the sample required to achieve a desired power and (B) estimate the power that can be achieved with a given sample size. In this work both features of the tool were used. The calculations (A and B) were carried out, each, twice as detailed below

1. The 'true' binary model is used to combine the alleles and obtain the 'true' genotype data. Those 'true' genotypes are then used in a linear predictor to generate the outcome data. The 'true' genotypes are subsequently used in the GLM fitted to assess the association. Thus, there is no misspecification since the 'true' underlying genetic model is used throughout the calculations; we refer to this as '*no misspecification*'.
2. The 'true' binary model is used to combine the alleles and obtain the 'true' genotypes data. Those 'true' genotypes are then used in a linear predictor to generate the outcome data but an additive model is used to combine the alleles and generate the genotypes in the fitted GLM. There is a misspecification because although the 'true' genetic model is used to generate the outcome data; an incorrect model (an additive rather than the 'true' binary model) is used in the GLM analysis. This is what we refer to as '*misspecification*'.

Overall a total of four analyses were carried out, two (with and without misspecification) for a binary outcome and two (with and without misspecification) for a continuous outcome. Each analysis was carried out with eight different minor allele frequencies (MAFs) to examine the effect of the misspecification error across different MAFs (see Table 1). This was done because the proportion of individuals in the study sample size whose genotypes were incorrectly assessed depends on the frequency of the minor allele which we considered to be the risk allele. It is reasonable to hypothesize that the magnitude of the error resulting from the misspecification of the underlying genetic model depends on the proportion of individuals whose genotype was incorrectly assessed and hence on the MAF. The prevalence of the binary outcome (e.g. disease) was set arbitrarily to 0.40.

For the binary outcome, the effect size of the SNP was set arbitrarily to an odds-ratio (OR) of 1.5 and for the continuous outcome it was set to 0.25. These values are in the range of magnitudes that one can reasonably expect in a candidate gene study. Both the outcome and the SNP were assumed to be measured without error: the binary outcome was simulated with sensitivity and specificity values of both 1. The continuous outcome was simulated with a reliability of 1 and the alleles used to construct the genotypes were simulated with sensitivity and specificity values of both 1. Hence, the only error introduced was the one resulting from the genetic model misspecification.

The input sample size was set to 1000 cases and 4000 controls for the association with a binary outcome and to 2500 subjects for the continuous outcome. The desired level of statistical power was set to 80%. In each analysis, we estimated simultaneously (A) the sample size required to achieving 80% power and (B) the power

achieved with the input sample size. Finally, we compared the output values (empirical sample size, empirical power, effect size and p-value) obtained when the 'true' model (binary) was used in the analysis to those obtained when the 'incorrect' model (additive) was used.

The values used to set the characteristics of the SNP and the outcome, are under **Table 1** and **2**.

Table 1:Minor allele frequency and effect size of the genetic determinant. Two calculations carried out one for a binary outcome (OR = 1.5) and one for a continuous outcome (effect size = 0.25).

Outcome	MAF	Effect Size
Binary	0.006, 0.007, 0.009, 0.02, 0.04, 0.06, 0.08, 0.1	1.5
Continuous	0.006, 0.007, 0.009, 0.02, 0.04, 0.06, 0.08, 0.1	0.25

Table 2: Prevalence of the binary outcome and distribution of the continuous outcome.

Outcome	Prevalence	Mean	SD
Binary	0.40	-	-
Continuous	-	0	1

2. Tabulated results of the analyses in the manuscript

Each of the tables in this document consist of two parts: Part (A) reports the sample sizes calculated with and without the misspecification error and the percentage increase in sample size required to compensate for the loss of power resulting from the misspecification error. Part (B) reports the estimated empirical power, OR, and p-value with and without the misspecification error.

2.1. Binary outcome

Table 3:(A) Sample size required to achieve the desired power of 80% with the true and the misspecified genetic model and (B) estimated empirical power, odds-ratio and p-value with the true model and with the misspecified genetic model when the outcome is a binary variable and for different MAFs.

(A) Number of cases required to achieve 80% power				
MAF	Binary SNP analysed as binary (CM)	Binary SNP analysed as additive (MM)	(MM-CM)/CM	SNP prevalence
0.006	18300	18391	0.50%	rare
0.007	16020	16043	0.14%	
0.009	10562	10598	0.34%	
0.020	5180	5220	0.77%	Low frequency
0.040	2843	2915	2.53%	
0.060	1916	1976	3.13%	common
0.080	1524	1596	4.72%	
0.100	1287	1359	5.59%	

(B) Power achieved with 1000 cases and 4000 controls					
	Statistical power		Estimated OR		SNP prevalence
MAF	Binary SNP analysed as binary	Binary SNP analysed as additive	Binary SNP analysed as binary	Binary SNP analysed as additive	
0.006	0.00	0.01	1.42	1.42	rare
0.007	0.01	0.01	1.41	1.41	
0.009	0.01	0.02	1.45	1.45	
0.020	0.06	0.09	1.43	1.42	Low frequency
0.040	0.17	0.30	1.42	1.40	
0.060	0.33	0.53	1.44	1.40	common
0.080	0.50	0.69	1.44	1.39	
0.100	0.61	0.78	1.44	1.38	

2.2. Quantitative outcome

Table 4: (A) Sample size required to achieve the desired power of 80% with the correct and the incorrect genetic model and (B) estimated empirical power, odds-ratio and p-value with the true model and with the misspecified genetic model when the outcome is a quantitative outcome and for different MAFs. The shaded dark grey, medium grey and light grey highlight the three SNP prevalence ranges, respectively, rare, moderately common and common.

**(A) Number of cases required to achieve 80% power**				
MAF	Binary SNP analysed as binary (CM)	Binary SNP analysed as additive (MM)	(MM-CM)/CM	SNP prevalence
0.006	32303	32606	0.94%	rare
0.007	25936	26134	0.76%	
0.009	22307	22489	0.82%	
0.020	9243	9335	1.00%	Low frequency
0.040	4855	4943	1.81%	
0.060	3480	3583	2.96%	common
0.080	2757	2862	3.81%	
0.100	2384	2511	5.33%	

**(B) Power achieved with 1000 cases and 4000 controls**					
MAF	Statistical power		Estimated OR		SNP prevalence
	Binary SNP analysed as binary	Binary SNP analysed as additive	Binary SNP analysed as binary	Binary SNP analysed as additive	
0.006	0.01	0.01	0.24	0.24	rare
0.007	0.01	0.01	0.25	0.25	
0.009	0.02	0.02	0.24	0.24	
0.020	0.09	0.09	0.25	0.25	Low frequency
0.040	0.32	0.30	0.25	0.24	
0.060	0.56	0.53	0.25	0.24	common
0.080	0.72	0.69	0.25	0.23	
0.100	0.81	0.78	0.25	0.22	

3. Impact of the misspecification error with larger MAFs

In the below results we show that the impact of the misspecification of the genetic model is larger with larger with more common SNPs indicating what we already reported in the main document and in the section above. For this analysis, the prevalence of the binary outcome (e.g. disease) was calculated using the Hardy-Weinberg Equilibrium (HWE) formula and an arbitrary penetrance of 70% (i.e. we assume that 70% of the individuals carrying the risk allele develop the disease). Below are the input parameters used for the results reported in the this section, with more frequent SNPs.

Table 5:Minor allele frequency and effect size of the genetic determinant. Two calculations carried out one for a binary outcome (OR = 1.5) and one for a continuous outcome (effect size = 0.25).

Outcome	MAF	Effect Size
Binary	0.025, 0.05, 0.1, 0.15, 0.2, 0.25, 0.3, 0.35	1.5
Continuous	0.025, 0.05, 0.1, 0.15, 0.2, 0.25, 0.3, 0.35	0.25

Table 6: Prevalence of the binary outcome and distribution of the continuous outcome.

Outcome	Prevalence	Mean	SD
Binary	0.035, 0.068, 0.133, 0.194, 0.252, 0.306, 0.357, 0.404	-	-
Continuous	-	0	1

3.1. Binary outcome

Table 7:(A) Sample size required to achieve the desired power of 80% with the true and the misspecified genetic model and (B) estimated empirical power, odds-ratio and p-value with the true model and with the misspecified genetic model when the outcome is a binary variable and for different MAFs.

**(A) Number of cases required to achieve 80% power**				
MAF	Binary SNP analysed as binary (CM)	Binary SNP analysed as additive (MM)	(MM-CM)/CM	SNP prevalence
0.025	7692	7793	1%	0.025 – 0.1
0.050	1750	1794	3%	
0.100	1095	1162	6%	
0.150	928	1009	9%	0.15 – 0.2
0.200	847	959	13%	
0.250	834	977	17%	0.25 – 0.35
0.300	844	1010	20%	
0.350	880	1100	25%	

**(B) Power achieved with 1000 cases and 4000 controls**					
	Statistical power		Estimated OR		
MAF	Binary SNP analysed as binary	Binary SNP analysed as additive	Binary SNP analysed as binary	Binary SNP analysed as additive	SNP prevalence
0.025	0.04	0.03	1.47	1.45	0.025 – 0.1
0.050	0.38	0.37	1.47	1.44	
0.100	0.73	0.69	1.47	1.41	
0.150	0.83	0.79	1.45	1.37	0.15 – 0.2
0.200	0.89	0.82	1.45	1.34	
0.250	0.90	0.82	1.44	1.31	0.25 – 0.35
0.300	0.88	0.78	1.44	1.29	
0.350	0.89	0.76	1.44	1.26	

3.2. Quantitative outcome

Table 8: (A) Sample size required to achieve the desired power of 80% with the correct and the incorrect genetic model and (B) estimated empirical power, odds-ratio and p-value with the true model and with the misspecified genetic model when the outcome is a quantitative outcome and for different MAFs. The shaded dark grey, medium grey and light grey highlight the three SNP prevalence ranges, respectively, rare, moderately common and common.

**(A) Number of cases required to achieve 80% power**				
MAF	Binary SNP analysed as binary (CM)	Binary SNP analysed as additive (MM)	(MM-CM)/CM	SNP prevalence
0.025	7822	7902	1%	0.025 – 0.1
0.050	4041	4162	3%	
0.100	2321	2454	6%	
0.150	1779	1937	9%	0.15 – 0.2
0.200	1548	1743	13%	
0.250	1484	1736	17%	0.25 – 0.35
0.300	1444	1756	22%	
0.350	1475	1886	28%	

**(B) Power achieved with 1000 cases and 4000 controls**					
	Statistical power		Estimated OR		
MAF	Binary SNP analysed as binary	Binary SNP analysed as additive	Binary SNP analysed as binary	Binary SNP analysed as additive	SNP prevalence
0.025	0.12	0.12	0.25	0.24	0.025 – 0.1
0.050	0.42	0.4	0.25	0.24	
0.100	0.85	0.80	0.25	0.23	
0.150	0.94	0.91	0.25	0.21	0.15 – 0.2
0.200	0.98	0.96	0.25	0.20	
0.250	0.99	0.96	0.25	0.19	0.25 – 0.35
0.300	0.99	0.96	0.25	0.17	
0.350	0.99	0.94	0.25	0.16	

4. Further considerations

One may argue that the detrimental effect of genetic model misspecification on power *does not matter* because: (1) the error resulting from the misspecification has little effect on power for rare SNPs because there are nearly no individuals carrying two copies of the risk allele when a SNP is rare and there is only an extremely limited number of people carrying one allele. In other words, there are virtually no individuals whose risks are underestimated. It follows that with or without the error there is not enough power in the first place because there are not enough cases. (2) For common SNPs, the model misspecification is responsible for a substantial loss of power but when a SNP is largely prevalent, there is always a large number of cases to sample from and in such settings, also the argument can be that with such frequencies a study will have sufficient power. Although it has been so far difficult to gather enough cases to study conditions determined by rare SNPs, the situation is now rapidly changing as more and more whole-genome sequencing is being undertaken, large cohorts are being set up and consortia that achieve very large sample sizes through the sharing of data are emerging [18-20]. These large repositories of genetic/genomic data will allow for associations studies involving rare genetic variants.

Most importantly our results show that the effect size is relatively not shrank for rare SNPs even when the underlying genetic model is misspecified; therefore, it is extremely important to reduce errors that cost power because if the study is enough powered one may derive robust inference from the effect size which is close to the true effect size. As for common SNPs, it is true that it might not be difficult to recruit enough cases to compensate for an eventual loss of power resulting from the source of error we explored in this work. However, this argument does not take into account the relatively large shrinkage of the effect size resulting from the misspecification of the underlying genetic model, as shown in our results.
